# Experiences of women awaiting cervical CANCER screening results from selected hospitals in Accra, Ghana

**DOI:** 10.1186/s12889-022-13874-7

**Published:** 2022-08-01

**Authors:** Evans Appiah Osei, Stella Appiah, Ezekiel Oti-Boadi, Dorothy Hammond, Dorothy Baffour Awuah, Awube Menlah, Isabella Garti, Michael Baidoo

**Affiliations:** 1 School of Nursing and Midwifery, Department of Midwifery, Valley View University, P.O. Box DT, 595 Oyibi, Ghana; 2 Head of the Department of Nursing, Valley View University, Box AF 595, Adentan, Accra Ghana; 3 School of Nursing and Midwifery, Valley View University, Oyibi, Ghana; 4 Korle-Bu Teaching Hospital, Accra, Ghana; 5 School of Nursing and Midwifery, Valley View University, Oyibi, Ghana; 6 School of Nursing and Midwifery, Valley View University, Oyibi, Ghana; 7 University of Charles Darwin, Darwin, Australia; 8 Valley View University, Oyibi, Ghana

**Keywords:** Awaiting, Cervical cancer, Experiences, Screening, Women

## Abstract

**Background:**

The rate at which cervical cancer is diagnosed among women worldwide is alarming, nevertheless, millions of women have never undergone cervical cancer screening, and many more with cervical cancer die prematurely without accessibility to quality healthcare or effective treatment. Women’s experiences following cervical cancer screening have not been extensively studied especially in advancing countries like Ghana. Hence, the researchers aim to explore the experiences of women awaiting cervical cancer results at selected hospitals in Accra.

**Methods:**

An exploratory-descriptive qualitative design was adopted to purposively sample 48 participants engaged in face-face in-depth interviews, which were audio-taped and transcribed verbatim after. The interviews were guided by semi-structured interviews.

**Findings:**

The findings revealed 3 themes and 10 subthemes. The themes were pre-screening experience, intra-screening experience, and post-screening experience. Participants narrated the challenges they face before the screening, during the screening, and as they waited for their results to get ready. Despite some challenges reported, most of the participants indicated that they were willing to come for a retesting if recommended.

**Conclusion:**

In conclusion, participants who have undergone CCS have several experiences that may either motivate or discourage them from subsequent screening. Being aware of such experiences could help the nurses address them in order to increase the interest of the women in CCS.

## Introduction

Every year, in the less industrialized countries, 83% of cervical cancer new cases and 85% of cervical cancer deaths occur [1]. The high mortality rate for cervical cancer in these countries is driven by limited access to cervical cancer screening, laboratory methods used to detect cervical cancer, and health-seeking behaviors [2]. It was also discovered that cervical cancer incidence rates increased with age and were higher for black women than white women [3]. In Ghana, cervical cancer is the most common cancer affecting women, with 50.5% attributed to the human papillomavirus (HPV) types 16 and 18, and is the cause of 16% of mortality due to cancer [4]. Consequently, some authors in Ghana found out that patient who was diagnosed late experience physical, psychological, social disruptions in their daily lives, which affected their quality of life [5].

The rate at which cervical cancer is diagnosed among women worldwide is alarming, nevertheless, millions of women have never undergone cervical cancer screening, and many more with cervical cancer die prematurely without accessibility to quality healthcare or effective treatment [6–8]. Studies conducted in South Africa and other low-income and middle-income countries revealed that a lack of adequate knowledge about cervical cancer and screening programs can detrimentally influence women’s likelihood of accessing such programs [9]. Additionally, evidence suggest lack of equipment and cervical cancer screening supplies in Sub-Saharan Africa [10].

In sub-Saharan African societies, the burden of cervical cancer is on the increase mainly due to inadequate provision and utilization of cervical cancer prevention services [11]. Several evidence-based strategies have been deployed to improve cervical cancer screening uptake without much success. The strategies include increasing awareness of cervical cancer screening, making cervical cancer screening available, and improving the attitude of cervical cancer screeners. Decreasing CC screening cost and provision of funding and improving the quality of screening are other strategies to help reduce CC incidence and improve CCS uptake [2, 10]. Patients go through varied challenges before, during, and following cervical cancer screening, however, satisfaction with service provision has not been adequately studied [11]. The challenges associated with cervical cancer screening include fear of being diagnosed with CC, and showing signs of post-traumatic stress disorder following diagnosis [12] Moreover, the anxieties associated with cervical cancer screening may be attributed to exposure to information on social media [13]^.^

Some experiences shared by participants of a study include long waiting times during the screening, non-adherence to follow-up of women with abnormal Pap smear, and unavailability of screening centers at the primary healthcare facilities [5]. It was also discovered that some women in Ghana experience distress whilst going for cervical cancer screening or awaiting their screening results [5]. This distress was found to be influenced by a lack of understanding about the smear test result ad having the fear that cancer will be detected. A study in Ghana, also ascertained that some women were embarrassed about exposing their vagina to strangers [14]. However, no study was found on the experiences of women awaiting cervical cancer screening, hence this study is to explore the experiences of women awaiting cervical cancer results at selected hospitals in Accra.

## Methods

### Research design

An exploratory-descriptive qualitative design was adopted in this study to understand the subjective experience as it relates to the social and psychological phenomenon of women [15]. The focus of the exploratory descriptive design is to gain insight and familiarity for later investigation.

### Target population

The study population comprises women who have undergone cervical cancer screening and presented their results at the Korle-Bu teaching hospital, Greater Accra Regional hospital, and 37-Military hospital, in Accra. Those who were excluded from the study were women who were yet to undergo screening, women with cervical cancer who are seriously sick, and those who were unwilling to partake in the study.

### Sampling technique

A purposive sampling technique was used to make sure that the selected participant was representative. This Strategy in which particular persons or events are selected deliberately to provide important information that cannot be denied from other choices [16]. Women who have undergone CCS and waiting for their screening results met the inclusion criteria. Hence, these women were contacted in the two selected hospitals and the purpose of the study was explained to them. Those who agreed to participate in this study were recruited and interviewed.

### Sample size

The sample size for this study was based on data saturation [16]. Saturation is the point in data collection where no new or relevant information is discovered concerning the constructed theory [17]. Saturation is a tool used for ensuring that adequate and quality data are collected to support the study [18]. The researcher, therefore, interviewed participants until no new information was retrieved. This was reached at the 48th participant.

### Data collection tool

A semi-structured interview guide designed by the researchers and pre-tested was used. The interview guide has 4 sections. The section has questions about participants’ socio-demographic characteristics whilst the other three sections collected data on participants’ experiences before the screening, during the screening and following the screening.

### Data collection procedure

Data collection was commenced after an ethical clearance had been obtained from the Dodowa Health Research Centre Institutional Review Board (DHRCIRB) with a protocol number DHRCIRB/052/05/21. A pilot study was conducted after using patients from a private hospital in Ghana. Following this, an introductory letter together with the ethical clearance was submitted to the administration of the Korle-Bu teaching hospital, Greater Accra regional hospital, and the 37 military hospitals to seek permission. A visit was paid to the Reproductive health department at the Obstetrics and gynecology units of various facilities where the study was to take place. The researchers then formally introduced themselves to the heads of the units and nurses, and enlightened them on the purpose of the study, the sample to be collected for the study.

Covid – 19 protocols were observed by both the interviewer and the participant. Hand sanitizers and facemask were available and supplied to all participants before the interview was conducted. The interviews were conducted after the participant consent form had been obtained and the purpose and procedure of the research had been explained to the client attending the clinic who met the inclusion and exclusion criteria. They were made to understand that they can decide whether or not to participate in the study. Forty-eight (48) in-depth face-face interviews were conducted in this study and took about 40–60 minutes each. Information was audiotaped during the interviews. The data collection lasted for 4 months.

### Data analysis

In this study, thematic content analysis was adapted since it is a good approach in trying to find out about the views, opinions, experiences, and values through the interview-guided transcript. The thematic content analysis process helps in reducing a large chunk of data into smaller fragments, by determining the presence of certain words, themes, or concepts within some qualitative data [19]. The researchers primarily acquainted themselves with the data by transcribing the audio recorder verbatim. The transcript was read and re-read to become familiar with what the data entails, paying specific attention to patterns that occur and submerging data to facilitate coding. In the second step, coding categories were determined which is based on the systematic structure to help make easier replication and improve reliability. The data was analyzed in thematic content within and across codes were performed to identify recurring themes whiles collating all identical and sorting diverse codes to form potential themes. Also, the code of content was determined by assigning a label to the text that has been analyzed and the text can be a word or a phrase in this phase [20]. This helps in the generation of themes and sub-themes.

### Methodological rigour

Four dimensions of rigour were proposed by some authors, namely, credibility, transferability, dependability, and confirmability in qualitative research to ensure the rigor and trustworthiness of the qualitative study [21]. The researchers ensured this accuracy by enlisting clients who gave informed consent and qualified using inclusion criteria for the study. The responses from the participant were confirmed at the end of the interview session before conclusions were drawn. In this study, transferability was applied by making sure that a detailed explanation of the methodology and guidelines were provided in all data collected.

## Results

### Socio-demographic characteristics of participants

The target populations of participants for the study were women who have undergone cervical cancer screening and were waiting for their results at selected hospitals in the Accra metropolis. The participants were between the ages of twenty-two [22] and sixty-three (63) and were selected during their weekly interviews. The study revealed that twenty-eight [23] were married, ten [10] were single, five [5] were widowed, and five [5] were divorced. Another interesting finding was that all the participants have given birth to at least one child. The language used for the data collection interview was English. Personals who conducted the screening were 15 (31%) male doctors, 12 female doctors (25%), 6 (13%) female nurses, and 15 (31%) midwives. 43 (90%) of women screened were first-timers whilst 5 (10%) reported that it was their second time screening for cervical cancer. The details have been illustrated in Table 1.

**Table 1:** Demographic Characteristics of Participants

Variable	frequency (*n* = 48)	Percent
**Age group**		
20–30	15	31%
31–40	10	21%
41–50	10	21%
> 50	13	27%
**Religion**		
Christian	38	79%
Muslim	8	17%
Traditional	2	4%
**Level of education**		
Primary	10	21%
Secondary	10	21%
Tertiary	28	58%
**Marital status**		
Single	10	21%
Married	28	58%
Divorced	5	10.5%
Widowed	5	10.5%
**Occupation**		
Government workers	18	38%
Private workers	10	21%
Students	6	13%
Self-employed	6	13%
Not working	8	15%
**Number of children**		
0	0	0%
1	8	16%
2	30	63%
≥ 3	10	21%
**Screening personnel**		
Male Doctors	15	31%
Female Doctors	12	25%
Midwives (Females)	15	31%
Nurses (Females)	6	13%
**Number of times screened**		
First-timers	43	90%
Second timers	5	10%
More than 2 times	0	0%

### Organization of themes and sub-themes

**Fig. 1:**
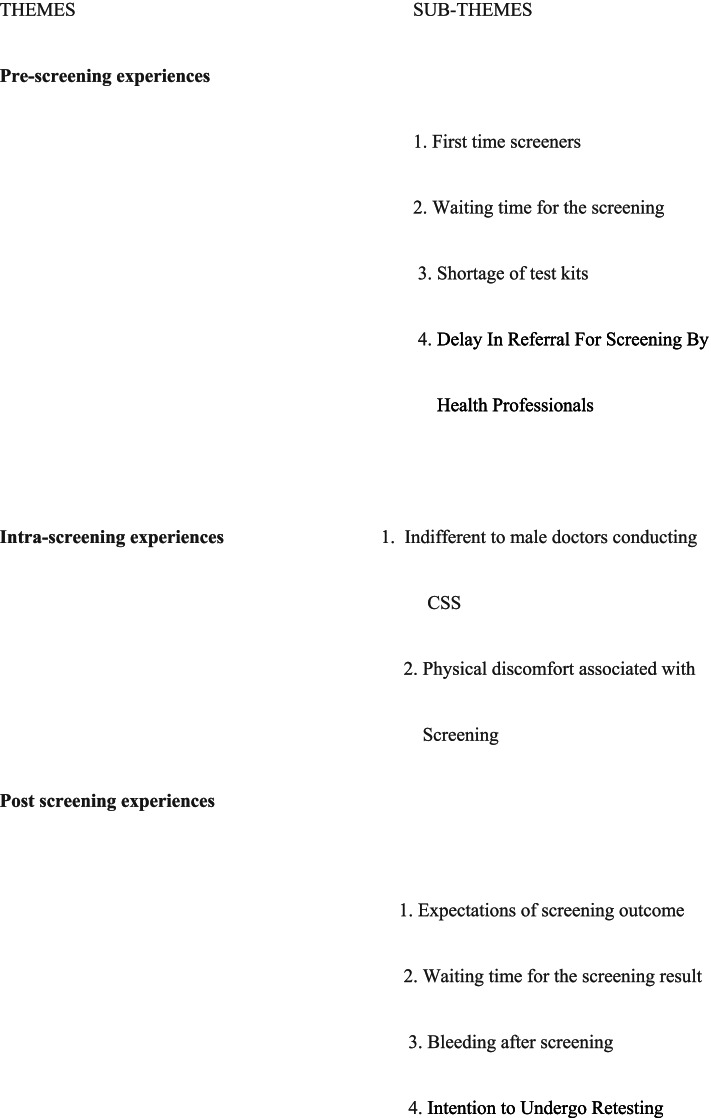
Themes, Emerging Themes and Sub-Themes

Three major themes were generated from the data. The major themes were grouped into subthemes and a total of ten [10] sub-themes were collated. To maintain anonymity, pseudonyms were used for each selected quote. The details have been presented in Fig. 1.

### Theme one: pre-screening experience

#### First time screeners

Most of the participants in this study (90%) were first-time screeners. Inadequate information for these first-timers and not knowing what to expect increased their fears and anxiety. First-time screeners in this study complained about the screening procedure as some participants revealed that it was uncomfortable.

“*It was my first time of screening and it was quite uncomfortable, you can’t imagine how I was nervous I came for the screening it was because I did not know much about how the screening was done but after going through I became less nervous”.(****P 48, 30 years).***

*“My heart was beating so fast when I presented last month for the screening since it was my first time, so I wanted them to do the screening fast for me to go home, but funny enough, they were delaying. (****P 1, 31 years).***

Other participants said they remain calm using deep breathing exercises and some relaxation techniques to help them cope with the procedure.

“*I have not screened for pap smear so that was my first time. I was much worried and had to be calm through some relaxation techniques and deep breathing the nurse showed me to perform because she said it will help me relax better since it was my first screening experience*”. (***P 46, 25).***

A participant who had come for her second screening said she was more relaxed as compared to the first one.

*“oo it went well. This is my second time screening. I already knew what to expect and how it is done so I was more confident and relaxed” (****P 43, 27 years).***

#### Waiting time for the screening

In Ghana, long waiting periods at hospital facilities tend to be a challenge faced by patients daily as this hinders most clients from going to the hospitals to seek treatment. According to the clients, the health care professionals showed little or no concern about their delays as they wait for the screening. These were some of the comments from respondents;

*“For the waiting … yes! You know our hospital system in Ghana. Everything you do you have to wait. I reported early around 7 am hoping it will be done early so I leave but now look at the time it’s 11 am and am now leaving. I don’t know why this happens but health service in Ghana needs to improve their services”. (****P2, 24 years).***

*“Oooohh … You can spend the whole day seating without being attended to. Am not surprised at all I spent 5 hours before the screening was done for me. You have to spend the whole day in the hospital anytime you come, so am used to them”. (****P24, 52 years).***

However few of the patients claimed that they didn’t have to wait long before the screening was conducted.

*“Ooh, I didn’t have to wait that long for the screening. Immediately I got here I spoke to the nurse at the reception and she made me fill out some forms and ushered me inside where the sample was taken so I didn’t have to sit and wait for that long. I think that’s because I had written down my name. I guess I didn’t have to wait”. (****P32, 45 years).***

*“I was surprised the process was so fast.* When I came, they quickly examined me and took me to the screening room, unlike my previous experience at the hospital. I came at 9 am and it is 10 now I am done with the screening” **(P 47, 35 years).**

#### Shortage of test kits

The shortage of test kits used to collect samples was detected as one of the factors that negatively influenced patient experiences. This was because some women who encountered this challenge were rescheduled their screening dates since the facility was in short supply of the test kit. In their account, majority of the participants (38) indicated their level of distress at the cost of transportation and difficulty in accessing the health facility.

*“I was asked to book for a later date when I came here some few weeks ago to conduct the test. The only reason I was given was the fact that they had run out of supply of the test kit. Where am coming from is quite far because I have to also pay that huge amount for the screening? I did not want to come again but finally today I came and they did it for me” (****P31, 49 years).***

*“Hmm, I was here last week Friday to do the screening but I was told to come back on Wednesday since they have run out of the test kit they use to take the sample. So I came back this past Wednesday to do the screening. I met two people who also came to do the screening and they had similar experiences but thanks to God we have all done it now*”. **(*P4, 28 years).***

#### Delay in referral for screening by health professionals

Delay in referral is one of the major challenges associated with the health profession. Most physicians delay referring patients for further examination or investigation. This may affect the prognosis of a cancer diagnosis. Immediate referrals to a specialist unit can reduce mortality incidence when adhered to. Most participants who came in late for screening reported that they time wasted time at various primary health facilities.

*“Hmm … I wished I had reported earlier by now I will be well and gone home. I didn’t why the doctor at the clinic delayed in giving me a referral for further examination because I kept on complaining about the pains and the discharges for more than 3month before I was given a referral”. (****P42, 29 years).***

*“Back in my hospital, the doctor gave me a referral letter after I have done several investigations. But I wasted time doing herbal medicine because I was scared of what will have happened to me. I stayed home with the referral for a month trying to raise funds for the hospital treatment. And had to come today with a worse situation, but finally, I am done”. (****P 40, 40 years).***

*“I remember all the tests I did and still have some of the reports here with me. These private hospitals will take all your monies when they know they can’t help before they will send you away. At first, I thought they are good only for me to realize they never really helped with my situation. How I wished I had come earlier”. (****P34, 37 years).***

### Theme two: intra-screening experience

#### Physical discomfort associated with screening

Despite well-organized screening programs effective in reducing the incidence of cervical cancer, physical discomfort associated with screening cannot be overlooked. The physical discomfort identified in this study among the participants during the screening was excessive sweating during the screening and frequent urination before the screening. The discomfort was observed to influence their reaction during the screening. There were varying views about the level of discomfort for each participant.

“*When I entered the room, I did not know what was happening to me. I had to excuse them to use the washroom. My heart just started beating fast. The nurse realized I was anxious too so she kept on reassuring me that everything will be well.” (****P35****, 23 years).*

*“When I was about to go inside for the procedure to be done, I started sweating profusely but the doctor did well as he spoke nicely with me which helped me to relax. Maybe because this test procedure is something new to me”. (****P36, 50 years).***

Few participants expressed that the speculum inserted was caused them pain and pressure in the lower abdomen during the procedure.

*“Yes... I experience some pains and pressure in my lower abdomen when the speculum was inserted into my vagina during the procedure. It was very uncomfortable for me. And was not so much worried because I sometimes have pains during sex” (****P 37, 37 years).***

#### Indifferent to male doctors conducting cervical Cancer screening

The healthcare profession is dominated by females, however, the number of male personnel’s kept increasing recently. Patient preference for specific gender attending to them in the various hospitals is becoming of much concern especially when their privacy will be invaded. In a situation like that, the patient normally prefers healthcare providers of the same gender. Interestingly, most of the participants in this study, (31%) reported that they were screened by male doctors, however, more than half of them (55%) revealed that they had no issue concerning being screened by males.

*“This wasn’t a big deal for me when I entered and saw a young doctor who was a male to examine me because this is not the first time a male physician was seeing my nakedness” (****P19, 26 years).***

*“I was very comfortable even though it was done by a male because all my siblings are males and as an old woman like me of 45years of age, what do I have to hide? I was not bothered”. (****P11, 38 years).***

Moreover, these participants narrated that, the male doctors were very caring and had no problems with them conducting the screening as compared to their female counterparts.

*“This is the second time I was doing the screening. The first one was done by a female but the male doctor was so nice to me by taking his time to talk to me and made sure I was prepared psychologically before beginning the screening”. (****P13, 26 years).***

“*The fact that I entered and saw a young male doctor made me at ease because you know when male touches you is different from a female touch so I was calm throughout the process” (****P 38, 51 years).***

On the contrary, participants reported that he was afraid of being in a room naked with a male doctor.

“*I have been raped before when I was 8years so I wasn’t comfortable at all being in a room alone with a male doctor, unlike the first time I did it, which was done by a female. Because of this I was sweating and he asked me to calm down and he is going to finish soon which made me a bit ok” (****P 37, 37 years).***

### Theme 3: post-screening experiences

#### Expectations of the screening outcome

Patients experiencing suggestive symptomatic disease of cervical cancer usually are in the state of looking forward to laboratory result outcomes and hoping for a negative result outcome. The majority of the participants were expecting the outcome to be negative though they were having symptoms suggestive of cervical cancer.

*“I reported to the doctor with severe bleeding and watery discharge and I was told it might be symptoms of cancer but I first have to do the screening. I am expecting the resulting outcome to be exactly what the doctor told me. Today I am here for my results, and I wish it comes out negative” (****P28, 30 years).***

*“Even though I was experiencing intermittent pains in my lower abdomen and some offensive vaginal discharges, I don’t want to believe the result will turn out positive. My expectations were very high and I can’t wait to see the outcome of my results at last.” (****P17, 43 years).***

Some participants had no expectations because they stated that they would accept whatever the outcome was.

*“It’s cool …*. *Don’t know what the outcome will be. Whether negative or positive am cool. Because I have already gone through hell”. (****P 44, 34 years).***

*“I do not know the outcome since it was a voluntary decision to conduct the text so if it’s positive fine and if it is negative I will accept it and go for treatment”.* ***(P13, 28 years).***

#### Waiting time for screening results

Cervical cancer screening results usually take longer for the result to be out. Many reasons may be accounted for the delay of the screening result. The participants claimed ones the procedure itself does not take long, they were hoping the report will be ready as soon as possible.

*“I was told the result will be out in two* [2] *weeks so I came here today but was told the result is not ready so have to leave and come back another time to check if it’s ready. The nurse gave me her number to call to find out if the result is ready before coming. Waiting for more than 2 weeks for a result which doesn’t even require 10 min to conduct the procedure is very bad” (****P42, 29 years).***

*“It took a while for my first result to be out. Me, I am a busy woman and I will always want fast results. But I know it will delay”.(****P 39, 47 years).***

Few participants commented that the waiting time for results increased their fears and stress.

*“Yes: I haven’t been myself lately. I just can’t help thinking over and over about it. I want to know what was causing me to bleed this much so am worried. it is very stressful whiles you wait for such a long time for the result to be out”. (****P38, 51 years).***

*“As for me If things delay I get worried. Was told I will be called when the result is out but it usually will take a few weeks to be ready.” (****P20, 33 years).***

#### Bleeding after screening

Some of the cervical cancer screeners came in with bleeding before the screening. This increased their chances of bleeding after the sample was taken. Those with heavy bleeding per vagina were asked to go back since it will interfere with the procedure. Some participants who experience slight bleeding after the procedure were detained and monitored for a while before being discharged home. This was depicted in the narration below;

*“Yes! I was bleeding that day but it wasn’t that much. I noticed some stains on my pad but it was very little. The bleeding was more after the test was done. The nurse suggested I lie down on the bed for a while before going home. She checked my BP constantly before she discharged me home”. (****P 46, 25 years).***

*“I noticed some bleeding after the procedure and I used about 4 sanitary pads after the procedure. The first pad was very soaked with blood and I changed it when I was still in the hospital. It later reduced so I used an additional 3 for the day. The nurse informed me that if I changed my sanitary more than 6times I should come back to the hospital”. (****P44, 26 years).***

#### Intention to undergo retesting

Retesting refers to a follow-up test that is usually done to determine if a person’s condition has improved or as a confirmation of a result. Patient with initial negative testing who needs retesting may not feel comfortable going for retesting due to fear that the results might change. Surprisingly, the majority of the participants in this study agreed to come for retesting.

*“Ooo. I will. It is all about my health so I don’t think I will have any problem repeating the test. I believe the doctor will explain further to me why a retest is to be done and once he can convince me, I will do it”.* ***(P7, 42 years).***

*“Ooo yes..... why not? Sometimes we repeat our lab test because the doctors will like the test to be done at specific laboratory centers known for quality standards. I have done several tests that I was asked to repeat due to that reason and it helped me to know the actual problem. So I will do a retest only if it won’t cause another bleeding.*” *. (****P14, 42 years).***

One participant cited that, she will not retest since she doesn’t have extra money to spend on another screening.

“*I will not do it. What happened to the first one I did and why do they want me to repeat it? Am not going to pay another kobo for a test. At least they will tell me why I have to do another test. I don’t have the money to be wasting on one test”. (****P5, 63 years).***

## Discussion

### First-time screeners

In this current study, most of the participants who were screened for the first time indicated that they experienced some level of anxiety coupled with discomfort from how the procedure will be done as a result of the little information they had about the CSS. Even though anxiety and discomfort were exhibited by some of the participants, few participants revealed that the psychological preparation given by healthcare professionals before the procedure made them relaxed towards the procedure. Education on CSS when given through various means will help increase adherence, reduce fears and anxiety, and encourage more women to screen. In accordance, 62.7% of study participants in the UK, cited pain/discomfort which they experienced previously or the potential fear of the pain or discomfort they will through in the process of the screening negatively influenced the decision to get screened [22].

### Waiting time for screening

With regards to the waiting time for CSS, it was revealed by participants of this current study that time-wasting was a norm in various facilities in the country which transcended to the CSS. The majority of the participants mentioned there were long queues when seeking healthcare including CSS which cause them to be delayed at the facility. The general attitude of healthcare providers about being time conscious was also lacking. What this means is that, if participants believe the procedure is time-wasting be it from inadequate health facilities or accessibility of the facility; most prospective clients are likely to lose interest in the screening which might lead to poor utilization of CSS. The time participant waited before being attended to by the health care provider had a significant relationship with satisfaction with cervical cancer screening. Similarly, a study in Malawi showed that the more time the women spent waiting for the service, the less satisfaction with the service received [24]. This revelation proved that waiting time is a major component of the patient experience which the current study also confirms.

### Shortage of test kits

The inconvenience created as a result of a shortage in the supply of test kits was encountered by some participants in this study. Due to the shortage of test kits, some of these women were rescheduled for the screening which was of much worry to them considering the distance and amount involved in the screening. The majority of the women studied 38 (79%) complained of how the lack of kits wasted their time and increased the cost of expenditure since they have to travel to and fro the facility on more than one occasion before finally getting tested and that it equally created undue stress. The findings of this current have been affirmed by previous study findings where participants indicated a lack of equipment and supplies for CCS in some Sub-Saharan African countries [10]. Hence these women are more likely to forgo the screening especially if they think they do not have any obvious health problems than going to waste their time. To overcome this the authors suggested the need to train more CC screening personnel, purchase more test kits and equipment for screening, and increase the number of screening centers [10].

### Delay in referral for screening by health professionals

It is generally observed that patients are detained for treatment until their condition becomes critical before they are referred for further management. Participants mentioned that they experienced delays in seeking facilities that had the appropriate services for cervical cancer screening for various reasons. Some expressed delays from the health institutions they initially sought care where they spent weeks and in some instances months before being finally referred to another facility but by the time the referral was given, symptoms had progressed. Others also attributed the delays to the refers centers to their own making since they were referred timely but refuse to go due to financial constraints This is supported by a survey done in Nepal where delays in referral to facilities for properly diagnosing and management complicated the patient’s condition as the disease progressed [25]. In addition, in Morocco, delay in reporting to the referral site was associated with the literacy level of the woman, the remoteness of where the patient was referred from to the referral site, and a patient who has not had cervical cancer screening in the past three years [26].

## Intra-screening experience

### Indifferent to male doctors conducting CCS

The findings from the study indicated that within the health profession, the number of male personnel keeps increasing and it was therefore not surprising that the majority of participants in this study had male doctors conducting their CSS. They further revealed that the gender of the health professionals was not of much concern to them as they were more concerned about getting screened and knowing their health status. Additionally, they claimed that most of the male doctors were caring so they had no fears or anxieties throughout the procedure. This implies that the health-seeking behavior for CSS among these women will be high since they were not bothered about who screened them. Hence, leads to the development of a good attitude towards CSS and in the long term results in early detection and treatment. In contrast, a study found that most of the university participants in Korea expressed a very strong dislike of having the screening conducted by a male doctor as they expressed feeling shame, fear, and distress they went through as a result of being screened by a male doctor [27]. Similarly, a previous study conducted in rural Ghana, found out some of the participants were quite embarrassed about exposing their private parts to a male health professional for screening [14].

### Physical discomfort associated with screening

Physical discomfort associated with the screening procedure is one of the vital findings from this current study. According to the participants, they experienced some level of anxieties and fears also manifested in their frequent use of the washroom, palpitations, and excessive sweating. Few also reported feeling pressure and pain in their vagina as the speculum was been inserted. This indicated that the psychological and physical effects of screening cannot be overlooked. Monitoring of vital signs and counseling of women before screening can serve as a form of assessment for participants who experienced physical discomfort. A previous study has revealed that the assessment of vital signs and level of pain was often considered physically significant as women reported feeling uncomfortable and painful during the screening [28]. It is therefore important to properly train and monitor healthcare providers who screen women for cervical cancer to ensure they give the best professional care to their patients as this will help manage their discomfort.

## Post-screening experience

### Expectations of screening outcome

Surprisingly, participants of this current study were hopeful that even when they were experiencing signs suggestive of cervical cancer, they were expecting a negative result. Some were also indifferent as they have been in pain for a while and just want to know the cause of their sickness to resolve their pain. Being hopeful could be viewed as something positive which could encourage them to go for the screening, however, they may be subjected to a great deal of psychological distress. if the results turn out to be positive since they are not expecting it. On the contrary, findings of a previous study revealed that fear of CCS results prevents many women from reporting for CCS [23].

### Waiting time for the screening result

How long it takes for the result from the screening to be available to the participants was seen to have an impact on the participants. Findings from this study highlighted that the expectations from the participants were that the results would not take long in being ready as the procedure itself was very short. But unfortunately, this was not the case as a significant number of the participants narrated that the results were not mostly ready at the time scheduled by the healthcare professionals, hence they have to be asked to go home and come another time when they report on the scheduled day and according to them, this heightened their anxieties. To buttress this finding is a established apprehension among participants due to long waiting hours for their results [9]. This current study agrees with a study done in South Africa where it realized the interval between waiting for the result from the screening had the women having anxiety about the possible outcome [9].

### Bleeding after screening

The current study findings revealed that the majority of the participants who were having vaginal bleeding before their screening cited that the screening induced further bleeding. This made them less anxious since they were conversant with it. However, they identified that they were managed appropriately following the screening. This finding agrees with the survey in India which indicated that vaginal discharge was the commonest complaint followed by inter-menstrual bleeding among women who were referred to do cervical cancer screening [29]. Similarly, the study findings aligned with a survey done in Ghana previously where participants who reported severe bleeding were eager to have the screening done and know the resulting outcome since it has affected their sexual activities and other lifestyles [30].

### Intention to undergo retesting

Retesting is something most people dislike doing-especially if their results are negative. However, those with positive results may be willing to do a retest hoping to get a change of results. Most of the women who took part in the study pointed out that they had no difficulties with having to undergo the screening again. The majority of the participants in this study agreed to come for a retesting if indicated by their doctor since they believe that it is an opportunity to learn more about cervical cancer, have their questions answered, and confirm their status. Adding on, some also stated they will go for retesting only if it is well explained to them the need to do retesting. However, few participants indicated an unwillingness to retest due to financial challenges. Hence, healthcare professionals should be more concerned about educating women on the need to do retesting after some time to motivate them. A study found that participants in Norway are more willing to be tested when it is fully explained on the test and is well understood^32^.

### Implications for study

The results of the study will be useful for other researchers working around women experiences during CCS. In addition, it will be useful for GHS and MOH to formulate policies to help ease anxieties and challenges faced prior to and during screening to increase CCS uptake.

## Conclusion

In conclusion, participants who have undergone CCS have several experiences that may either motivate or discourage them from subsequent screening. Being aware of such experiences could help the nurses address them in order to increase the interest of the women in CCS. This will also help other women to know what to expect during the screening and following the screening to help reduce their anxieties prior to screening.

## Data Availability

All data supporting this manuscript have been made available. All data generated or analyzed during this study are included in this published article.
